# The factors influencing the accuracy of pre‐operative endoscopic ultrasonography assessment in endoscopic treatments for gastrointestinal tumors

**DOI:** 10.1002/cam4.5305

**Published:** 2022-09-29

**Authors:** Yan Zhao, Mudan Ren, Ai Jia, Juan Zhang, Shuying Wang, Qian Zhao, Guohong Cai, Shuixiang He

**Affiliations:** ^1^ Department of Gastroenterology First Affiliated Hospital of Xi'an Jiaotong University Xi'an China; ^2^ Department of Nuclear Medicine Xijing Hospital, Fourth Military Medical University Xi'an China

**Keywords:** diagnostic accuracy, endoscopic submucosal dissection, endoscopic ultrasonography, gastrointestinal superficial tumors, pre‐operative assessments

## Abstract

**Background:**

This retrospective study aimed to evaluate the factors influencing the accuracy of Endoscopic Ultrasonography (EUS) as a preoperative assessment for gastrointestinal tumors.

**Methods:**

A total of 261 patients with 264 gastrointestinal tumors were enrolled in the study. The parameters of the gastrointestinal lesions examined under EUS and their pathology were recorded and analyzed.

**Results:**

The accuracy of EUS for detecting intramucosal lesions and subepithelial lesions (SELs) were 83.6% and 91.4%, respectively. One hundred and ninety‐four (73.5%) lesions originated from the mucous layer, as determined by pre‐operation EUS examinations. The accuracy of EUS in predicting the correct T stage for intramucosal lesions in the gastric region, esophagus, and colorectum was 77%, 71.8%, and 84.6%, respectively. According to the Paris endoscopic classification, the distribution of macroscopic patterns was different between the EUS‐pathology conformity and nonconformity groups (*p* = 0.018). In the nonconformity group, 48.6% of erosive lesions were classified as 0‐IIc, 0‐IIa + IIc, 0‐IIc + IIa or 0‐III macroscopic patterns compared with 26% patients in the conformity group (*p* = 0.025). Univariate analyses demonstrated that ulcerative lesions (OR = 7.516, 95% Confidence Interval [CI] 2.574–21.952, *p* < 0.001), location at the cardia of the stomach (OR = 3.619, 95%CI 1.076–12.168, *p* = 0.038), malignant tumor (OR = 2.920, 95%CI 1.339–6.368, *p* = 0.007) were significantly associated with EUS inaccuracy. Multivariate logistic regression analyses showed that ulcer was an independent risk factor associated with EUS inaccuracy, with odds ratios of 5.094 (95% CI: 1.641–15.807, *p* = 0.005).

**Conclusions:**

Our findings suggested that EUS is a reliable and easy‐to‐use diagnostic tool in decision‐making regarding appropriate endoscopic treatment for gastrointestinal tumors. However, the diagnostic accuracy of EUS appeared questionable in the presence of ulceration.

## Graphical abstract

Preoperative staging of superficial gastrointestinal tumors is crucial for choosing the most appropriate treatment. Our findings suggested that endoscopic ultrasonography (EUS) was a reliable and easy‐to‐use diagnostic tool in decision‐making regarding appropriate endoscopic treatment for gastrointestinal tumors. However, the diagnostic accuracy of EUS appeared questionable in the presence of ulceration.

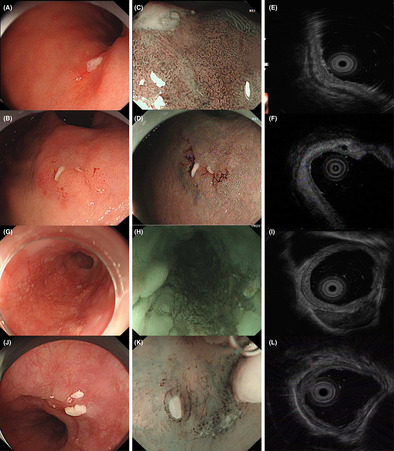

## 1 INTRODUCTION

Gastrointestinal superficial tumors are defined as lesions with invasion limited to the mucosa or submucosa.^1^ Endoscopic submucosal dissection (ESD) is an innovative treatment for gastrointestinal superficial neoplastic lesions without lymph node metastases.^2^ Previous studies have shown that ESD is safe and effective for tumors originating from the mucosal and submucosal layers.^3^ Endoscopic submucosal excavation (ESE), endoscopic full‐thickness resection (EFTR), and submucosal tunneling endoscopic resection (STER), which are procedures derived from ESD, were used for subepithelial lesions (SELs) according to tumor growth pattern and invasion depth. Compared to surgical treatment, endoscopic treatments have the advantages of minimal complications, shorter hospital stays, and preservation of gastrointestinal function.^4^ However, it is suggested that the risk of lymph node metastasis increases with increasing depth of tumor invasion.^5^ The aim of our clinical work was to avoid inappropriate excision of a carcinoma with deep submucosal invasion and unnecessary resection. Therefore, it is crucial to determine the accurate staging and origin of gastrointestinal tumors before endoscopic treatment.

Since the 1980s, endoscopic ultrasonography (EUS) has emerged as a promising diagnostic examination for superficial gastrointestinal neoplastic lesions.^6^ It is a practicable examination to diagnose the degree of gastrointestinal tumor invasion and origination by evaluating the lesions' originating layer, echo level, and internal echo pattern. Particularly high‐frequency catheter probes become a handy and potentially powerful diagnostic tool in clinical practice.^7^ Kwee et al. conducted a systematic review and reported that EUS, computed tomography (CT), and magnetic resonance imaging (MRI) achieved similar results, but EUS still remained the first‐choice in preoperative evaluation of T staging of gastric cancer because more experience has been gained with EUS.^8^ Although EUS showed excellent diagnostic value for gastrointestinal neoplastic lesions, it remains unclear whether EUS can accurately predict the characteristics and invasion depth of lesions.^9^ Considering that EUS relies on the operators' experience to some degree, it may underestimate or overestimate the stages of lesions.^10^

Therefore, we conducted this large‐scale retrospective cohort single‐center study to evaluate the value of EUS as a preoperative assessment for endoscopic treatment procedures. We also aimed to determine the factors that affect the diagnostic accuracy of EUS in gastrointestinal superficial tumors.

## 2 METHODS

This was a retrospective cohort study conducted in northwestern China between January 2018 and December 2020. The inclusion criteria for the study population were as follow: gastrointestinal tumors; treated with ESD, ESE, EFTR or STER; undergone EUS examination before resection. All consecutive patients who met these criteria were included unless they had any of the following conditions: without complete recording data; failed endoscopic treatments and conversion to surgery. Informed written consent was waived because of the retrospective nature of the study. The following data were collected: age, sex, tumor location (mucosal or submucosal tumor), tumor size, Paris endoscopic classification, and pathological examination results.

### 2.1 ESD and EUS procedures

ESD procedures were performed by three experienced doctors (average experience >600 ESD procedures accumulatively). ESD was performed using standard endoscopy (GIF‐H290; Olympus, Japan), and a transparent cap was attached to the tip of the endoscope.^11^ The circumferential incision procedure was performed with an insulated‐tip knife (KD‐611 L; Olympus, Tokyo, Japan) and a HOOK‐knife (KD‐620LR; Olympus, Tokyo, Japan). EFTR was chosen for those located in the deep muscularis propria (MP) layer with extraluminal growth patterns. It was also used for the ones for which complete dissection with ESE was difficult.^12^ In STER procedure a mucosal dissection proximal is created to the lesion to allow a submucosal tunnel.^13^ The wound was then closed with multiple clips in a “side‐to‐center” manner or with the aid of an endoloop (MAJ‐339; Olympus, Japan).^14^

EUS examinations were performed by two experienced endo‐sonographers with an average experience of more than 200 EUS procedures each year. EUS examinations with a radial‐scanning echo‐endoscope (GIF‐Q260J; Olympus, Japan) were performed in all patients to access the layers of origin and invasion before endoscopic treatment. The flexible, high‐frequency catheter probes that can be introduced through the working channel of endoscopy was used in the procedure. Miniprobes as a supplement to echoendoscopes with radial or longitudinal scanners which can provide detailed images of both the structures of the intestinal wall and a considerable volume of the surrounding organs.

The macroscopic pattern was defined according to the update Paris endoscopic classification of superficial neoplastic lesions: pedunculated (0‐Ip), sessile (0‐Is), slightly elevated (0‐IIa), completely flat (0‐IIb), slightly depressed (0‐IIc), elevated and depressed types (0‐IIc + IIa, 0‐IIa + IIc), ulcer (0‐III) and excavated and depressed types (0‐IIc + III, 0‐III + IIc).^15^, ^16^

### 2.2 Pathology examination

The removed tissue specimens were analyzed by hematoxylin–eosin staining and immunohistochemically with immunohistochemical staining. Pathology data, including histological tumor type, infiltration depth, and tumor differentiation grade, were collected from the medical record system. The malignant tumors refer to adenocarcinoma or squamous cell carcinoma were confirmed by pathology examination.

The histopathological findings were compared with the results of the EUS assessments after resection. The invasion depth was classified according to the layer of tumor invasion: m means the lesions were confined to the mucous layer; sm1 means invasion into the submucosa<500 μm from the muscularis mucosae; and sm2 means invasion into the submucosa ≥500 μm from the muscularis mucosae. For lesions in the colon and esophagus, the cutoff limits are 1000 μm and 200 μm, respectively.^15^

### 2.3 Statistical analysis

Continuous variables are presented as mean and standard deviation (SD). Categorical variables were expressed as frequencies and percentages. The difference of distribution between EUS and histological staging conformity and nonconformity groups in relation to the location, macroscopic pattern, lesion size and curative resection rate was assessed using the chi‐squared test or Fisher's exact test. Logistic regression and random forest models were used to assess the accuracy of EUS and influencing factors. Ten‐folds cross validation and receiver operating characteristic curve (ROC) were used to assess the effect of models. Univariate and multivariate analyses were conducted to identify the factors associated with EUS accuracy. Odds ratios (ORs) with 95% confidence intervals (CIs) were calculated. All tests of significance were two‐tailed, and a *p* value of <0.05, was regarded as significant. Analyses were performed using SPSS (version 17.0) and R software packages (version 4.0.3).

## 3 RESULTS

### 3.1 Characteristic of overall patients and lesions

Overall, this study consecutively included 261 patients with 264 gastrointestinal tumors, wherein 152 were men, and the mean age was 58.7 ± 11.9 years (range 16–85 years). All patients underwent pre‐procedure EUS examinations. The median time between EUS and endoscopic resection was 5.3 ± 3.6 days (range 2–28 days). Patient characteristics and clinicopathological features of the lesions were summarized in Table 1. The most frequent locations of lesions were the gastric (*n* = 103), esophagus (*n* = 82), followed by the colorectum (*n* = 71) and duodenum (*n* = 8). The mean tumor diameter was 1.2 ± 0.9 cm. All lesions were treated with ESD, ESE, EFTR, or STER. The mean procedure time was 45 ± 21 min (range, 15–120 min). In total the successful curative en bloc resection rate was 95.1%. The lateral margins were positive in two gastric lesions, five esophageal lesions and one colorectal lesion. The vertical margins were positive in four gastric lesions, three esophageal lesions and one colorectal lesion. The complete resection rate in stomach, esophagus, colorectum, and duodenum were 94.3%, 89.7%, 95%, and 100%, respectively. The endoscopic resection related complications including bleeding and perforation. Bleeding during resection was almost inevitable, and hemostatic forceps was very effective for controlling the bleeding. In our study, there was no case that required transfusion, interventional radiology or surgery due to bleeding. The postoperative delayed bleeding rate was 2.3%. The perforation rate for ESD was 3.1% and was successfully controlled with endoscopic clip closure.

**TABLE 1:** The baseline information of the 261 patients with 264 lesions

Characteristic	*n*
Male	152 (57.8%)
Age, years, mean ± SD	58.7 ± 11.9
Tumor size, cm	
≤1.0	156
1.0–2.0	67
≥2.0	41
Mean lesion size (cm) ± SD	1.2 ± 0.9
Operative time, min, mean ± SD	45 ± 21 min (rang 15–120 min)
Location	
Esophagus	82
Rectum	38
Gastric antrum	34
Gastric body	28
Gastric fundus	17
Cardia	14
Gastric angle	10
Ascending colon	10
Transverse colon	9
Duodenum	8
Sigmoid colon	7
Ileocecal junction	5
Descending colon	2
Ulcerative findings	
No	248
Yes	16
Origination of lesions by EUS	
Mucosa	194
Subepithelial	70
Treatment methods	
ESD	213
ESE	40
EFTR	6
STER	5

Abbreviations: EFTR, endoscopic full thickness resection; ESD, endoscopic submucosal dissection; ESE, submucosal excavation; EUS, Endoscopic ultrasonography; SD, standard deviation; SMT, submuocsal tumors; STER, submucosal tunneling endoscopic resection.

### 3.2 The overall accuracy of EUS for lesions in mucous layer

**FIGURE 1:**
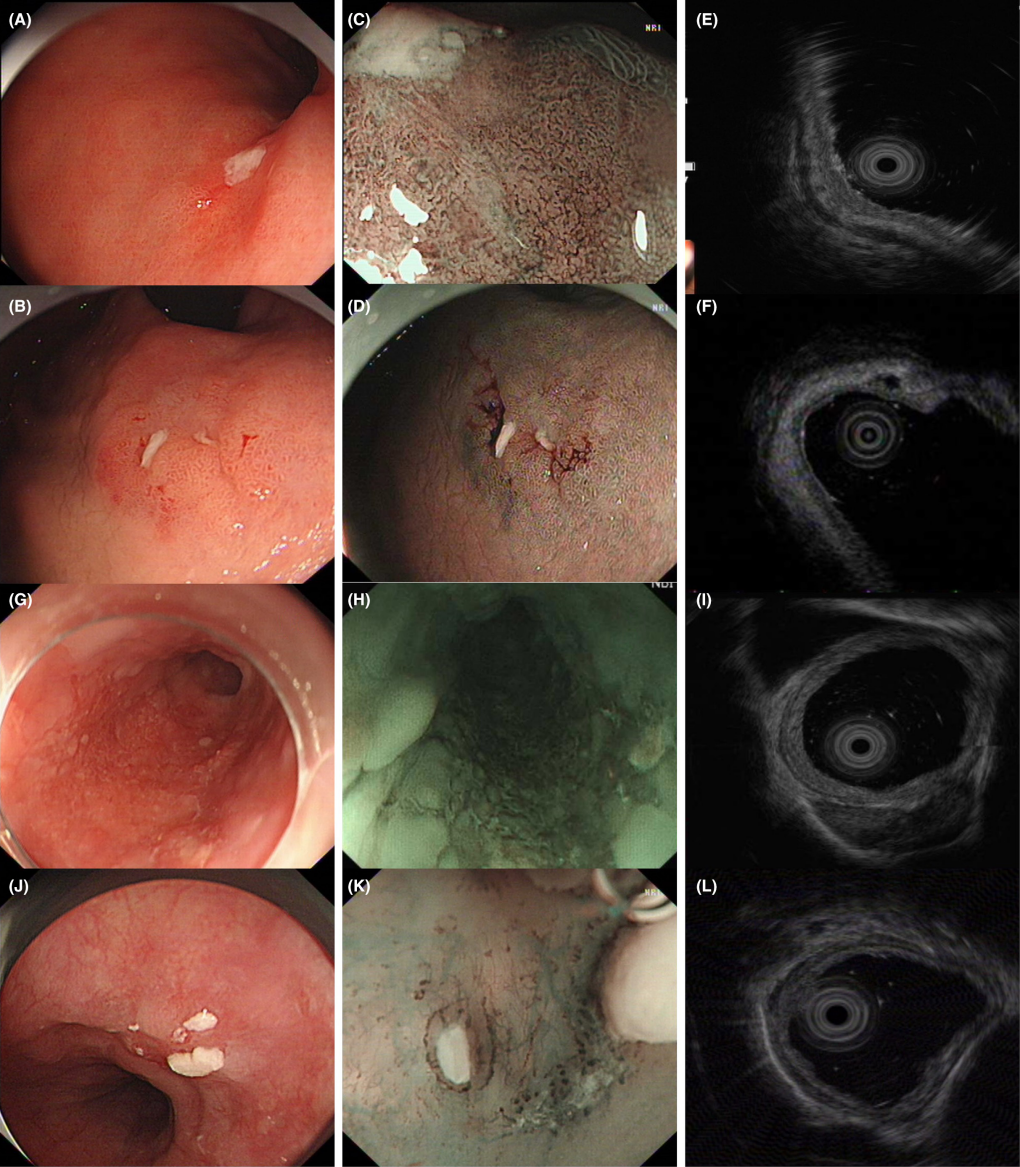
The cases in the EUS‐pathology nonconformity group. (A) and (B) two ulcerative lesions with pT1b‐SM2 at the cardia of stomach; (C) and (D) the NBI images of the lesions; (E) and (F) the EUS images of the lesions showed that the structure was complete and they were misjudged as T1a. (G) one flat lesion with pT1b‐SM1 at the esophagus; (H) the NBI image of the lesion; (I) the EUS image of the lesion showed that it was misjudged as T1a; (J) one lesion at the esophagus with pT1a‐LPM (lamina propria mucosa); (K) the NBI image of the lesion; (L) the EUS image of the lesion showed that the structure was incomplete and it was over‐staged as T1b.

One hundred and ninety‐four (73.5%) lesions originated from the mucous layer, as determined by pre‐ESD EUS examinations. In total, the diagnostic accuracy for predicting the correct T stage in gastrointestinal tumors was 83.6% in the test set in logistic regression model (Table 2). Here we showed fours cases with understaged or overstaged estimation by EUS which were located in the cardia of the stomach and esophagus in Figure 1. As shown in Table 3, 159 of 194 lesions were in the conformity group and 35 lesions belonged to the nonconformity group. According to the Paris endoscopic classification, the distribution of macroscopic patterns was different between the nonconformity and conformity groups (*p* = 0.018). In the nonconformity group, 48.6% of erosive lesions were classified as 0‐IIc, 0‐IIa + IIc, 0‐IIc + IIa or 0‐III macroscopic patterns compared with 26.4% patients in the conformity group (*p* = 0.025). The rates of ulcerative lesions with 0‐III patterns were higher in the nonconformity group than in the conformity group (*p* < 0.001).

**TABLE 2:** The diagnosis accuracy of EUS in the test set according to ten‐folds cross validation

Location	Logistic regression model		Random forest model	
	Accuracy	Kappa	Accuracy	Kappa
Overall	83.63%	0.23	84.07%	0.18
Stomach	77.02%	0.23	80.06%	0.15
Esophagus	71.76%	0.04	79.14%	0.13
Colorectum	84.62%	0.21	92.14%	0.26

Abbreviation: EUS, endoscopic ultrasonography.

**TABLE 3:** Comparison of EUS and histological staging conformity and nonconformity groups

	Conformity group (*n* = 159)	Inconformity group (*n* = 35)	*p*
Location of lesions			<0.001
Esophagus	45 (28.%)	13 (37%)	
Gastric	54 (34%)	16 (45.7%)	
Duodenum	5 (3.1%)	1 (2.9%)	
Colorectum	55 (34.%)	5 (1%)	
Macroscopic pattern			0.018
0‐Ip	5 (3.1%)	0	
0‐Is	57 (35.8%)	9 (25.7%)	
0‐IIa	27 (17.0%)	4 (11.4%)	
0‐IIb	27 (17.0%)	5 (14.3%)	
0‐IIa + IIb	1 (0.6%)	0	
0‐IIc	28 (17.6%)	4 (11.4%)	
0‐IIa + IIc	5 (3.1%)	4 (11.%)	
0‐IIc + IIa	2 (1.3%)	0	
0‐III	7 (4.4%)	9 (25.7%)	
Lesion size, cm, mean ± SD	1.8 ± 1.0	1.8 ± 1.1	0.868
Curative resection rate	152 (95%)	30 (88.2%)	0.229

Abbreviation: EUS, endoscopic ultrasonography.

Univariate logistic regression analyses demonstrated that ulcerative lesions (OR = 7.516, 95%CI 2.574–21.952, *p* < 0.001), location at the cardia of the stomach (OR = 3.619, 95%CI 1.076–12.168, *p* = 0.038), malignant tumor (OR = 2.920, 95%CI 1.339–6.368, *p* = 0.007) were significantly associated with EUS inaccuracy. Multivariate logistic regression analyses showed that ulcer was an independent risk factor associated with EUS inaccuracy, with odds ratios of 5.094 (95%CI 1.641–15.807, *p* = 0.005) (Table 4).

**TABLE 4:** Logistic regression analysis for factors associated with the accuracy of EUS

Variable	Univariate analysis			Multivariate analysis		
	OR	95% CI	*p*	OR	95% CI	*p*
Sex (male vs. female)	1.885	0.828–4.293	0.131			
Age (≥60 vs. <60 years)	0.836	0.452–1.546	0.568			
Erosion (with vs. without)	1.646	0.761–3.559	0.205			
Ulcer (with vs. without)	7.516	2.574–21.952	<0.001	5.094	1.641–15.807	0.005
Macroscopic pattern (uneven vs. flat)	1.282	0.457–3.596	0.636			
Lesions size (2 vs. ≤2 cm)	0.964	0.457–2.034	0.924			
Location (cardia of stomach vs. other)	3.619	1.076–12.168	0.038	1.889	0.485–7.361	0.36
Histologic type (malignant tumor vs. other)	2.92	1.339–6.368	0.007	2.118	0.925–4.850	0.36

Abbreviations: CI, Confidence Interval; EUS, Endoscopic ultrasonography; OR, odds ratio.

In addition, the overall diagnostic accuracy in the random forest model was 84.1%. The area under the ROC (AUC) was 0.700 (95%CI 0.567–0.812) with the sensitivity of 40% and the specificity of 100% (Figure S1). According to the importance of characteristics in the random forest model, the top five important influencing factors were lesions size, ulcer, histologic type, sex and location (Table S1).

### 3.3 The accuracy of EUS in gastric lesions

Seventy lesions were located in the gastric mucosa. On invasion depth assessment by EUS, the numbers of T1a and T1b lesions were 58 and 12, respectively. After the ESD procedure, the pathological examinations showed that 65 lesions were pT1a‐M, 4 were pT1‐SM2, and 1 was pT2‐MP. Table 5 shows the EUS results in comparison with histopathological results for T staging. The overall accuracy of EUS in predicting the correct T stage was 77% in logistic regression model (Table 2). A total of 11 lesions with pT1a stage were over‐staged as having T1b lesions on EUS (15.7%). In contrast, five lesions with more deep infiltration were downstaged as having T1a or T1b lesions by EUS (7.1%). Univariate logistic regression analyses demonstrated that ulcerative lesions (OR = 7.727, 95%CI 1.603–37.242, *p* = 0.011), location at the cardia of the stomach (OR = 4.029, 95%CI 1.113–14.583, *p* = 0.034) were significantly associated with EUS inaccuracy. Multivariate logistic regression analyses showed that ulcer was an independent risk factor associated with EUS inaccuracy, with odds ratios of 5.767 (95%CI 1.115–29.820, *p* = 0.037) (Table S2).

**TABLE 5:** Comparison of EUS and histological T staging in 70 gastric lesions

EUS staging, *n*	Histological staging, *n*			Total, *n*	correct
	pT1a‐M	pT1b‐SM2	pT2‐MP		
EUS T1a	54	4	0	58	77.10%
EUS T1b	11	0	1	12	0.00%
Total	65	4	1	70	77.10%
Overstaged					15.70%
Understaged					7.10%

Abbreviation: EUS, endoscopic ultrasonography.

In addition, the diagnostic accuracy of EUS in gastric lesions in the random forest model was 80.1%. The AUC was 0.714 (95%CI 0.478–0.887) with the sensitivity of 42.9% and the specificity of 100% (Figure S1). According to the importance of characteristics in the random forest model, the top five important influencing factors were lesions size, ulcer, sex, histologic type, and macroscopic pattern (Table S3).

### 3.4 The accuracy of EUS in esophageal lesions

Fifty‐eight lesions were located in the esophagus. Detailed information is shown in Table 6. On invasion depth assessment by EUS, the numbers of T1a and T1b were 46 and 12, respectively. The overall accuracy of EUS in predicting the correct T stage was 71.8% in logistic regression model (Table 2). A total of eight lesions with pT1a stage were over‐staged as pT1b lesions by EUS (15.5%). In contrast, five lesions with more deep infiltration were downstaged as T1a lesions by EUS (10.3%). In addition, there were 2, 39 and 17 lesions which were located at upper third, middle third, and lower third of esophagus with the EUS accuracy rate of 50%, 76.9%, and 82.4%, respectively. And the difference was not statistically significant (*p* = 0.490). Univariate logistic regression analyses demonstrated that ulcerative lesion was associated with EUS inaccuracy but failed to reach statistical significance with odds ratios of 6.450 (95%CI 0.949–43.861, *p* = 0.057) (Table S4).

**TABLE 6:** Comparison of EUS and histological T staging in 58 esophagus lesions

EUS staging, *n*	Histological staging, *n*					Total, *n*	correct
	pT1a‐EP	pT1a‐LMP	pT1a‐MM	pT1b‐SM1	pT1b‐SM2		
EUS T1a	24	14	3	2	3	45	70.70%
EUS T1b	1	5	2	1	3	1	6.9%
Total	25	1	5	3	5	5	77.6%
Overstaged							1%
Understaged							8.60%

Abbreviation: EUS, endoscopic ultrasonography.

In addition, the diagnostic accuracy of EUS in esophageal lesions in the random forest model was 79.1%. The AUC was 0.688 (95% CI 0.430–0.881) with the sensitivity of 50% and the specificity of 87.5% (Figure S1). According to the importance of characteristics in the random forest model, the top five important influencing factors were lesions size, ulcer, sex, age and histologic type (Table S5).

### 3.5 The accuracy of EUS in colorectal lesions

Sixty lesions were located in the colorectum. The diagnostic accuracy of EUS was compared with that of histological staging after ESD (Table 7). The overall accuracy of EUS in predicting the correct T stage was 84.6% in logistic regression model (Table 2). A total of four lesion with pT1a cancer was over‐staged as having pT1b lesions on EUS. On the contrary, one lesion with pT1b‐SM2 stage was downstaged as having T1a by EUS. Univariate logistic regression analyses demonstrated that ulcerative lesions (OR = 36, 95%CI 2.5–518.371, *p* = 0.008) and histologic type (OR = 7.667, 95%CI 1.117–52.637, *p* = 0.038) were significantly associated with EUS inaccuracy. Multivariate logistic regression analyses showed that ulcer was an independent risk factor associated with EUS inaccuracy, with odds ratios of 21.858 (95%CI 1.275–374.648, *p* = 0.033) (Table S6).

**TABLE 7:** Comparison of EUS and histological T staging in 60 colorectal lesions

EUS staging, *n*	Histological staging, *n*				Total, *n*	correct
	pT1a‐M	pT1a‐LMP	pT1a‐MM	pT1b‐SM2		
EUS T1a	51	0	4	1	56	91.70%
EUS T1b	3	1	0	0	4	0.00%
Total	54	1	4	1	60	91.7%
Overstaged						6.70%
Understaged						1.70%

Abbreviations: EUS, endoscopic ultrasonography.

In addition, the diagnostic accuracy of EUS in colorectal lesions in the random forest model was 92.1%. The AUC was 0.750 (95%CI 0.494–0.920) with the sensitivity of 50% and the specificity of 100% (Figure S1). According to the importance of characteristics in the random forest model, the top five important influencing factors were lesions size, histologic type, erosion, age, and sex (Table S7).

### 3.6 The accuracy of EUS in duodenal lesions

Six lesions were located in the duodenum. One lesion with pT1a‐M were over‐staged as having pT1b on EUS. The logistic regression and random forest models were not performed due to the small sample size.

### 3.7 The accuracy of EUS for SELs

Seventy lesions were identified as SELs on pre‐ESD EUS examinations. The final histopathological tests showed that there were 34 leiomyomas, nine gastrointestinal stromal tumors (GISTs), four tubular adenomas, four cases with chronic inflammation of mucosa, nine neuroendocrine tumors (NETs), three lipomyomas, four ectopic pancreas, one fibroma, one granulomatous inflammation, and one thrombus organization. The accuracy of EUS for diagnosing SELs was 91.4%. In these lesions, two lesions were considered as SELs by pre‐ESD EUS, but the final pathology results showed that they were tubular adenomas. One esophageal leiomyoma was erroneously diagnosed as calcification on EUS. One colonic inflammatory granuloma was evaluated as an SELs from the proper muscle by EUS. One ectopic pancreas was considered a thickened proper muscle under EUS. One lesion of the thrombus organization was erroneously considered as a leiomyoma on EUS.

## 4 DISCUSSION

Preoperative staging of gastrointestinal tumors is crucial for choosing the most appropriate treatment. EUS is a handy tool to visualize gastrointestinal lesions in different layers.^17^ Previous studies have shown that EUS can be used to assess the locoregional staging of gastrointestinal lesions.^18^ However, it remains controversial whether EUS is efficacious in determining the tumor origin and depth of invasion before treatment. In the guidelines by Japan Gastroenterological Endoscopy Society Guidelines Committee of ESD/EMR for esophageal cancer, the opinion that “EUS should not be used as standard procedure because there are no studies with high level of evidence” is weakly recommend.^19^ In addition, in the guidelines for ESD and endoscopic mucosal resection for early gastric cancer, it is suggested that EUS may be useful as an additional diagnostic modality.^3^ With the background of still unsatisfactory and complicated classification systems, EUS is still considered as an easy‐to‐use and complemental tool.^20^ Further studies are needed to determine which type of lesion is necessary to undergo EUS examination before treatment. It is important to analyze the accuracy of EUS and investigate its related influencing factors. We conducted this large‐scale cohort study to compare the accuracy of EUS with pathological examination of gastrointestinal lesions. In the present study, we reported that the overall accuracy of EUS for assessing the correct T stages of gastrointestinal mucosal lesions and SELs was 82.5% and 91.4%, respectively. The strengths of this study were its broad eligibility criteria. We included not only the mucosal lesions determined by EUS but also the SELs determined by EUS. We analyzed the lesions both from the upper and lower gastrointestinal tracts and compared the differences in EUS accuracy in various locations.

Previous studies have reported accuracies of approximately 60% to 90% for staging invasion depth (mucosa vs. submucosa) of EUS. Lee et al. retrospectively enrolled 393 lesions, wherein the results showed that the overall accuracy of EUS was 66.7%. They found that EUS had more frequent overestimation than conventional endoscopy.^21^ A study by Yanai et al. showed that the accuracy of EUS could reach 71% and is expected to compensate for the understating of lesions with submucosal invasion.^22^ Okada et al. also reported that the diagnostic accuracy of EUS in identifying lesions meeting the expanded indication criteria for ESD could reach as high as 87.8%.^18^ In our study, the diagnostic accuracy in predicting the correct T stage in the gastrointestinal tract was 83%. The overall accuracy was slightly higher than that of previous reports. This may be because of the increasing experience of endo‐sonographers and improved equipment quality of EUS year by year.

In terms of the factors associated with EUS accuracy, Hizawa et al. reported that the accuracy was lower in cases with lesions located in the upper third of the stomach, ulcer lesions, and those measuring more than 2 cm.^23^ In Choi's study, which enrolled 955 patients, the accuracy of EUS was lower in cases with lesions in the upper third of the stomach, tumor diameter more than 1.0 cm, presence of ulceration, submucosal invasion, and undifferentiated histology.^9^ Okada et al. suggested that ulceration and large tumors were associated with an incorrect diagnosis of tumor invasion depth by EUS.^18^ Thosani et al. conducted a meta‐analysis and reported that the sensitivity of EUS was 85% for T1a staging and 86% for T1b.^24^ Consistent with these previous reports, the univariate logistic regression analyses in our study revealed that ulcerative lesions, location at the cardia of the stomach, and malignant tumors were significantly associated with EUS accuracy.^9^, ^18^, ^23^ Although the multivariate logistic regression analyses showed that ulcer was the independent factor that affected the accuracy of EUS, the effect of location and the degree of malignancy should not be neglected.

There are several other possible explanations for this misdiagnosis of EUS. Firstly, one reason is that the inflammatory changes surrounding the tumor are difficult to discriminate from the tumor itself, which may result in overstaging of the infiltration depth.^17^ It has been suggested that 10%–30% of early gastric cancers have ulceration with accompanying fibrosis, which is seen as a hypoechoic lesion on EUS, similar to tumor invasion. This discrepancy is due to the additional echoes produced by the interfaces between different histologic layers. These ultrasound artifacts can cause a misevaluation of the depth of cancer invasion. Secondly, the different degrees of difficulty of EUS operation at various locations may affect its accuracy. In our study, there was no difference of EUS accuracy in various segmentations of esophagus. The cardia site is particularly important because it is difficult for operators to keep the probe in the water. Thirdly, to avoid the sedation‐related complications of EUS, all EUS examinations were completed while awake in our center. Under these circumstances, the degree of patient tolerance is another important factor that may affect the evaluation of EUS. In our study, the accuracy of EUS in the colorectum was higher than that in the gastric and esophagus. This result may be associated with the better tolerance of endoscopy in colorectum than that in upper gastrointestinal tract.

In addition, the Paris Classification, an international standard for endoscopic classification of gastrointestinal neoplastic lesions, helps us to predict the feasibility and curability of ESD for lesions.^25^, ^26^ Previous studies have shown that the different types of Paris classification are correlated with the invasion depth into the submucosa. And it is also correlated with the risk of nodal metastases.^27^ Paris 0‐I, 0‐IIc, and 0‐III were considered to have a higher risk of submucosal invasion compared with 0‐IIa and 0‐IIb lesions. However, data are scarce about the reproducibility of Paris Classification among different endoscopists. Our reports suggest that 0‐III ulcerative lesions are associated with EUS inaccuracy.

There were some limitations to this study. First, EUS was not examined by one operator, which means there was possible objective bias, although they were all experienced endo‐sonographers. Second, because this was a retrospective cohort study, the lack of a control group prevented us from accurately assessing the EUS value.

## 5 CONCLUSION

Although EUS has well‐known shortcomings, it is still the reliable and easy‐to‐use diagnostic tool for locoregional endoscopic treatments for gastrointestinal tumors. The results of our study support that EUS can aid in decision‐making regarding appropriate treatment. However, in the presence of ulceration the accuracy of EUS was unsatisfactory.

## AUTHOR CONTRIBUTIONS

**Yan Zhao:** Conceptualization (lead); formal analysis (lead); funding acquisition (lead); investigation (equal); methodology (equal); writing – original draft (lead); writing – review and editing (lead). **Mudan Ren:** Data curation (lead); project administration (equal); resources (lead); writing – original draft (equal). **Ai Jia:** Investigation (equal); project administration (equal); resources (equal); supervision (equal); writing – original draft (equal). **Juan Zhang:** Investigation (equal); resources (equal); validation (supporting); writing – original draft (supporting). **Shuying Wang:** Resources (supporting); supervision (supporting); writing – original draft (supporting). **Qian Zhao:** Investigation (supporting); resources (supporting); writing – original draft (supporting). **Guohong Cai:** Data curation (equal); methodology (equal); software (lead); writing – original draft (equal); writing – review and editing (lead). **Shuixiang He:** Conceptualization (equal); funding acquisition (equal); project administration (lead); writing – original draft (equal); writing – review and editing (lead).

## FUNDING INFORMATION

This study was supported by National Natural Science Foundation of China (Program No. 81900489), Natural Science Basic Research program of Shaanxi (Program No. 2022JM‐456) and Key Research and Development Program of Shaanxi (No.2021ZDLSF02‐06).

Data generated or analyzed during the study are available from the corresponding author by request.

## CONFLICT OF INTEREST

The authors have declared no conflicts of interest.

## ETHICS APPROVAL

The study was approved by the ethics committees and the requirement for written informed consent was waived because of the study's retrospective nature.

## Supporting information


Appendix S1

## Data Availability

We guarantee that the data mentioned in the article are completely accurate and the manuscript is original research that has not been published or is not under consideration elsewhere.
