# Supplementary material for: The factors influencing the accuracy of pre‐operative endoscopic ultrasonography assessment in endoscopic treatments for gastrointestinal tumors
Source: Cancer Med. 2022 Sep 29;12(4):4321–31. doi: 10.1002/cam4.5305 (PMC9972141; doi:10.1002/cam4.5305)
Supplement: Supplementary file 1 — Appendix S1 [file CAM4-12-4321-s001.docx]

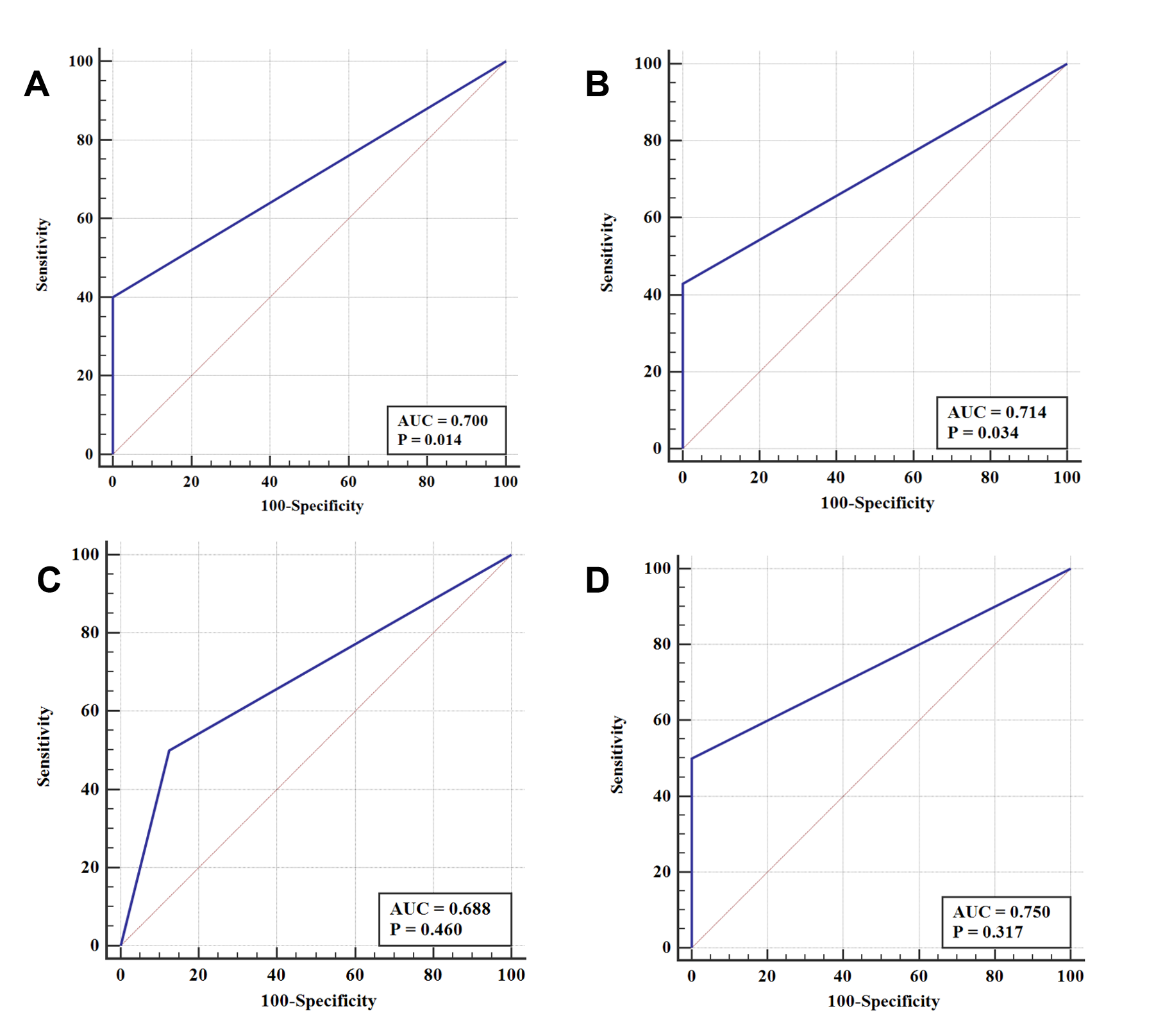


**Supplementary Figure 1**. The ROC curves in the test sets of random forest models in different tumor locations: A. overall lesions; B. gastric lesions; C. Esophageal lesions; D. Colorectal lesions.

Abbreviations: ROC, receiver operating characteristic curve.

| Supplementary table 1. the rank of importance of influencing factors in overall lesions in random forest models | | |
| --- | --- | --- |
| Variable | Mean Decrease Accuracy | Mean Decrease Gini |
| Sex | 0.51 | 2.01 |
| Age | -0.95 | 1.60 |
| Erosion | 0.44 | 1.39 |
| Ulcer | 7.45 | 3.69 |
| Macroscopic pattern | 1.63 | 0.82 |
| Lesions size | -2.11 | 6.13 |
| Histologic type | 1.63 | 2.13 |
| Location | 1.31 | 1.79 |

| Supplementary table 2. Logistic regression analysis for risk factors associated with the inaccuracy of EUS for gastric lesions. | | | | | | |
| --- | --- | --- | --- | --- | --- | --- |
| Variable | Univariate analysis | | | Multivariate analysis | | |
|  | OR | 95% CI | P | OR | 95% CI | P |
| Sex (male vs. female) | 1.765 | 0.501-6.216 | 0.377 |  |  |  |
| Age (≥60 vs. <60 years) | 0.862 | 0.282-2.633 | 0.794 |  |  |  |
| Ulcer (with vs. without) | 7.727 | 1.603-37.242 | 0.011 | 5.767 | 1.115-29.820 | 0.037 |
| Macroscopic pattern (uneven vs. flat) | 1.217 | 0.231-6.409 | 0.816 |  |  |  |
| Lesions size (2 vs. ≤2cm) | 1.011 | 0.562-1.816 | 0.972 |  |  |  |
| Location (cardia of stomach vs. other) | 4.029 | 1.113-14.583 | 0.034 | 2.852 | 0.709-11.482 | 0.14 |
| Histologic type (adenocarcinoma vs. other) | 1.491 | 0.485-4.586 | 0.485 |  |  |  |
| EUS, Endoscopic ultrasonography; OR, odds ratio; CI, Confidence Interval. | | | | | | |

| Supplementary table 3. the rank of importance of influencing factors in gastric lesions in random forest models | | |
| --- | --- | --- |
| Variable | Mean Decrease Accuracy | Mean Decrease Gini |
| Sex | 0.22 | 1.41 |
| Age | 0.47 | 0.38 |
| Erosion | -1.72 | 0.72 |
| Ulcer | 1.04 | 1.53 |
| Macroscopic pattern | -0.60 | 1.05 |
| Lesions size | 1.55 | 2.49 |
| Histologic type | -0.52 | 1.22 |
| Location | 1.17 | 0.81 |

| Supplementary table 4. Logistic regression analysis for risk factors associated with the inaccuracy of EUS for esophageal lesions. | | | | | | |
| --- | --- | --- | --- | --- | --- | --- |
| Variable | Univariate analysis | | | Multivariate analysis | | |
|  | OR | 95% CI | P | OR | 95% CI | P |
| Sex (male vs. female) | 1.839 | 0.441-7.664 | 0.403 |  |  |  |
| Age (≥60 vs. <60 years) | 0.834 | 0.379-1.835 | 0.651 |  |  |  |
| Ulcer (with vs. without) | 6.45 | 0.949-43.861 | 0.057 | 6.45 | 0.949-43.861 | 0.057 |
| Macroscopic pattern (uneven vs. flat) | 2.667 | 0.646-11.009 | 0.175 |  |  |  |
| Lesions size (2 vs. ≤2cm) | 1.094 | 0.685-1.749 | 0.706 |  |  |  |
| Histologic type (Squamous cell carcinoma vs. other) | 3.883 | 0.452-33.339 | 0.216 |  |  |  |
| EUS, Endoscopic ultrasonography; OR, odds ratio; CI, Confidence Interval. | | | | | | |

| Supplementary table 5. The rank of importance of influencing factors in esophageal lesions in random forest models | | |
| --- | --- | --- |
| Variable | Mean Decrease Accuracy | Mean Decrease Gini |
| Sex | 1.20 | 1.11 |
| Age | 1.56 | 0.76 |
| Erosion | 1.92 | 0.67 |
| Ulcer | 3.05 | 2.87 |
| Macroscopic pattern | 1.57 | 0.11 |
| Lesions size | 1.96 | 3.56 |
| Histologic type | 0.36 | 0.71 |

| Supplementary table 6. Logistic regression analysis for risk factors associated with the inaccuracy of EUS for colorectal lesions. | | | | | | |
| --- | --- | --- | --- | --- | --- | --- |
| Variable | Univariate analysis | | | Multivariate analysis | | |
|  | OR | 95% CI | P | OR | 95% CI | P |
| Sex (male vs. female) | 1.25 | 0.193-8.080 | 0.815 |  |  |  |
| Age (≥60 vs. <60 years) | 0.857 | 0.132-5.571 | 0.872 |  |  |  |
| Ulcer (with vs. without) | 36 | 2.5-518.371 | 0.008 | 21.858 | 1.275-374.648 | 0.033 |
| Lesions size (2 vs. ≤2cm) | 1.036 | 0.325-3.301 | 0.953 |  |  |  |
| Histologic type (adenocarcinoma vs. other) | 7.667 | 1.117-52.637 | 0.038 | 4.83 | 0.555-42.068 | 0.154 |
| EUS, Endoscopic ultrasonography; OR, odds ratio; CI, Confidence Interval. | | | | | | |

| Supplementary table 7. The rank of importance of influencing factors in colorectal lesions in random forest models | | |
| --- | --- | --- |
| Variable | Mean Decrease Accuracy | Mean Decrease Gini |
| Sex | -0.85 | 0.24 |
| Age | 1.97 | 0.48 |
| Erosion | 0.00 | 0.78 |
| Ulcer | 0.00 | 0.00 |
| Macroscopic pattern | 0.00 | 0.00 |
| Lesions size | -1.01 | 1.10 |
| Histologic type | -0.05 | 0.81 |
